# Premature thymic functional senescence is a hallmark of childhood acute lymphoblastic leukemia survivorship

**DOI:** 10.1038/s41408-024-01071-1

**Published:** 2024-06-13

**Authors:** Tibila Kientega, Sophie Marcoux, Jessica Bourbonnais, Jade Montpetit, Maxime Caru, Guillaume B. Cardin, Nathalie Arbour, Valérie Marcil, Daniel Curnier, Caroline Laverdière, Daniel Sinnett, Francis Rodier

**Affiliations:** 1 Centre de recherche du Centre hospitalier de l’Université de Montréal (CHUM), Montréal, QC Canada; 2 Institut du cancer de Montréal, Montréal, QC Canada; 3 Université Laval, Département de médecine sociale et préventive, Québec, QC Canada; 4 Centre de recherche du Centre hospitalier universitaire de Québec—Université Laval, Québec, QC Canada; 5 Centre de recherche Azrieli du CHU Sainte-Justine, Montréal, QC Canada; 6 Department of Pediatrics, Division of Hematology and Oncology, Penn State College of Medicine, Hershey, PA USA; 7 Université de Montréal, Département de Neurosciences, Montréal, QC Canada; 8 Université de Montréal, Département de Nutrition, Montréal, QC Canada; 9 Université de Montréal, Faculté de médecine, École de kinésiologie et des sciences de l’activité physique, Laboratoire de physiopathologie de l’exercice (LPEX), Montréal, QC Canada; 10 Université de Montréal, Département de Pédiatrie, Montréal, QC Canada; 11 Université de Montréal, Département de Radiologie, radio-oncologie et médecine nucléaire, Montréal, QC Canada

**Keywords:** Acute lymphocytic leukaemia, Risk factors, Lymphopoiesis, Paediatrics

## Abstract

Childhood acute lymphoblastic leukemia (cALL) survivors suffer early-onset chronic diseases classically associated with aging. Normal aging is accompanied by organ dysfunctions, including immunological ones. We hypothesize that thymic immunosenescence occurs in cALL survivors and that its severity may correlate with early-onset chronic diseases. The PETALE study is a cALL survivor cohort with an extensive cardiovascular and metabolic evaluation. The thymic immunosenescence biomarker, signal joint T-cell receptor excision circles (TREC), was evaluated and was highly correlated with age in healthy participants (*n* = 281) and cALL survivors (*n* = 248). We observed a systematic thymic immunoage accentuation in each cALL survivor compared to controls ranging from 5.9 to 88.3 years. The immunoage gain was independent of age at diagnosis and treatment modalities and was more severe for females. Thymic aging was associated with several pathophysiological parameters, was greater in survivors suffering from metabolic syndrome, but there was no significant association with global physical condition. The decrease in TREC was independent from blood cell counts, which were normal, suggesting a segmental aging of the thymic compartment. Indeed, increased plasmatic T cell regulatory cytokines IL-6, IL-7 and GM-CSF accompanied high immunoage gain. Our data reveal that cALL or its treatment trigger a rapid immunoage gain followed by further gradual thymic immunosenescence, similar to normal aging. This leads to an enduring shift in accentuated immunoage compared to chronological age. Thus, accentuated thymic immunosenescence is a hallmark of cALL survivorship and TREC levels could be useful immunosenescence biomarkers to help monitoring the health of cancer survivors.

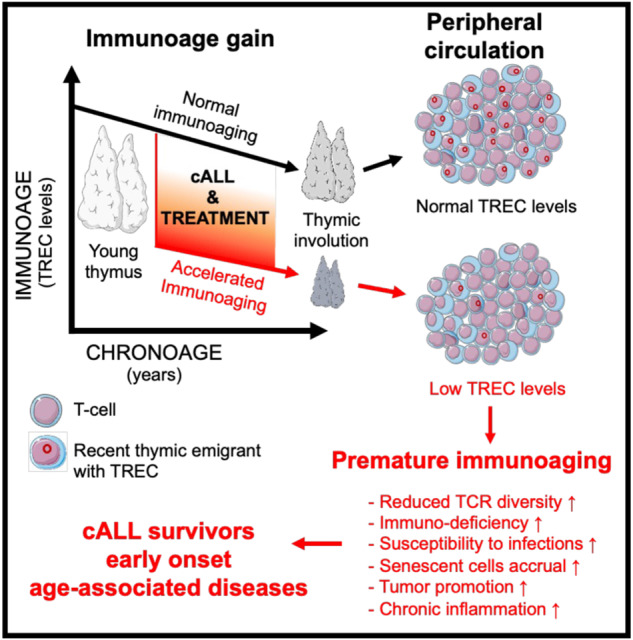

## Introduction

Immunological aging, referred to as immunoaging, is an umbrella term encompassing immunosenescence and inflammaging [1]. Inflammaging is characterized by the deregulation of numerous microenvironment factors including interferon gamma (IFN-γ), interleukins (IL-6, IL-7, IL-12, IL-15, IL-4), growth factors (bFGF), and Granulocyte-Macrophage Colony-Stimulating Factor (GM-CSF) [1, 2]. In the T-cell compartment, normal immunoaging is also associated with thymic involution, which leads to reduced recent thymic emigrants (RTE), reduced immune repertoire diversity, and amplified oligo-clonal expansion of educated memory cells [1, 3]. The signal joint-T cell receptor excision circles (TREC) in peripheral T-cells are a by-product of T-cell receptor rearrangement in the thymus [4]. TREC levels inversely correlate with chronological age in healthy humans and decrease steadily by approximately 4.9% per year [3, 5, 6]. TREC levels correlate with aging blood biomarkers and leukocytes telomere length and can be used in forensics to evaluate the age of suspects using blood samples [7–9]. Given the correlation between thymic output and thymic atrophy, a consequence of aging and immunosenescence, measuring TREC levels within T cell populations could serve as an accessible and effective means of quantifying immunosenescence [4, 10]. In turn, this could indirectly allow for the measurement of premature aging and early-onset age-related chronic diseases.

Despite improved 5-year survival rates of >90% for lymphoid leukemias, long term childhood acute lymphoblastic leukemia (cALL) survivorship is associated with multiple late adverse effects [11–13]. Sequelaes include age-related phenotypes such as hormonal abnormalities, frailty, cardiovascular diseases, chronic inflammation, metabolic syndrome and many other preventable chronic diseases [12, 14–17]. These early-onset diseases invoke premature aging syndrome [15, 18]. Due to their exposure to chemotherapy and radiotherapy, cALL survivors suffer extensive DNA damage, senescent cells accumulation, shortened leukocyte telomere length and a decrease in the numbers of all thymocyte and naïve T cells [14, 19–22].

cALL survivors are individually affected to different extents regarding premature chronic health incidence, thus biomarkers allowing the screening of patients at higher risk who would benefit from more intensive and personalized follow-up care are needed. Chronological age, measured by the time elapsed since birth, is a poor indicator of an individual’s actual health status [23, 24]. A biological age biomarker would be invaluable as a surrogate endpoint of health/fitness in prematurely aged population studies [24]. Biological age can be assessed using frailty indices or by biological age biomarkers including DNA methylation and cell senescence-associated phenotypes such as p16^INK4A^ expression, telomeres length and MCP1 expression [25–30]. However, frailty assessment tools and definitions are numerous and mostly qualitative, p16^INK4A^ assessments require well-preserved biopsied tissues, DNA methylation measure is expensive and time-consuming, while telomeres length heterogeneity among individuals makes it an unreliable individual measure of aging [25, 26, 30]. In this study, we tested whether the levels of the thymic aging biomarker TREC, which is easily measured from fresh or frozen blood samples, can reliably assess biological aging in cALL survivors. Further, we verified whether higher immunoage gains at the individual level were associated with early-onset chronic diseases in the PETALE cohort of long-term cALL survivors.

## Material and methods

### Study population

This study compared 248 cALL survivors from the PETALE cohort to 281 healthy controls. The PETALE study is a multidisciplinary research initiative with the goal of comprehensively examinig long-term adverse effects and identifying associated predictive biomarkers in cALL survivors [13]. Patients in the PETALE cohort were 0–18 years old at the time of cALL diagnosis and were treated at the Sainte-Justine University Health Center (CHUSJ) between 1987 and 2010. Treatment regimens followed the Dana Farber Cancer Institute (DFCI) protocols 87-01, 91-01, 95-01, 00-01 or 05-01, and depended on the participant’s risk group, whether they had a standard or high risk of relapse [31]. All cALL survivors received moderate to high doses of doxorubicin (41–472 mg/m^2) and total doses of corticosteroids ranging from 2135 to 24,901 mg/m^2. Among the participants who received a high dose of doxorubicin, 27.8% also received dexrazoxane. Additionally, some of these participants underwent age-dependent cranial irradiation (12–20 Gy). Exclusion criteria were being less than five years posttreatment at the time of study, refractory or relapsed disease or having received an hematopoietic stem cell graft [13]. Healthy participant cohort included individuals aged 19–40 years old (institutional cohort from the Centre Hospitalier de l’Université de Montréal) and 40–69 years old (province of Quebec [CANADA] study cohort CARTaGENE, (https://www.cartagene.qc.ca/index.html)). The CARTaGENE cohort made up of men and women randomly selected and reflects the general healthy population from Quebec areas, where 70% of the province’s population resides. Due to the age limitation of the CARTaGENE cohort, we recruited additional healthy participants, age-matched to the PETALE cohort, from the CHUM comprising students in training, workers, or visitors. Both the control CHUM and CARTaGENE datasets are combined, considering age- and sex-specific TREC levels, to calculate the immunoage (in years) in cALL. This study was approved by the Institutional Ethics Review Committee (#19.035 and #2013-3607) and conducted in accordance with the Declaration of Helsinki. All participants or participant’s guardian provided written informed consent.

### TREC quantification

TREC levels were assessed in peripheral blood mononuclear cells or whole blood samples. Copy number standards and DNA samples underwent preamplification before conducting triplex quantitative TaqMan™ PCR amplification of TREC, CD3, and unrearranged VD-J. This was carried out using the StepOne Plus™ real-time system (Applied Biosystems, California, USA) with the following protocol: 50 °C for 2 min, 95 °C for 10 min, followed by 40 cycles of 95 °C for 15 s and 60 °C for 1 min. The TREC value was estimated as the ratio of TREC to 100,000 T cells (Supplemental Methods).

### Pathophysiologic parameters

Outcomes and health status from cALL survivors were evaluated at the PETALE I follow-up appointment, during which blood samples were collected. We examined established health issues suggestive of premature aging or age related-diseases to compare with thymic aging. Physical deconditioning, abnormal 6-minute walk test (6MWT), weight loss, and metabolic syndrome were used as aging or frailty indicators within our cALL survivor cohort [15, 16, 32]. Metabolic syndrome was defined according to the International Diabetes Federation criteria and assessed in all participants. The criteria were abnormal waist circumference, high triglycerides, low HDL-cholesterol, high blood pressure ( ≥ 130 mm Hg systolic or ≥85 mm Hg diastolic), and high glucose or known type 2 diabetes mellitus [33]. DHEAS, LH, testosterone, or estradiol levels were measured using immunofluorometric assay at CHUSJ. The body fat percentage was measured by dual energy x-ray absorptiometry as described previously [34]. The 6MWT was performed twice in a 30-meter hallway according to a previously described standardized protocol [32]. Participants’ oxygen consumption was measured through a maximal cardiopulmonary exercise test (CPET) using the previously described McMaster incremental cycle protocol [35]. VO2max and 6MWT results from participants were converted to age- and sex-specific percentiles. Abnormal performance in 6MWT was defined as participants below the lower normative threshold for age and sex of the 95th percentile. Abnormal CPET or deconditioning was defined as survivors whose percentage of measured versus predicted vO2 peak was less than 100%. The plasmatic proteins IL-4, IL-6, IL-7, IL-12p70, IL-15, bFGF, GM-CSF, and INFγ were quantitatively measured in cALL participants plasma using multiplex ELISA (Mesoscale discovery V-PLEX Human Biomarker 40-plex, MESO SCALE DIAGNOSTICS, Maryland, US).

### Statistical analysis

Normality of the data was assessed using the Kolmogorov–Smirnov normality test. Parametric variables were analyzed using t-tests, Welch’s t-test, ROC curves and one-way analysis of variance, and nonparametric variables were analyzed using the Mann–Whitney U-test. ROC curve is used to evaluate if accentuated thymic aging could reveal chronic health condition occurrence. Linear and logistic multiple regression models were constructed using a stepwise forward approach. For the linear regression model, we initially employed various individual factors as independent variables, which included chronological age, sex, age at diagnosis, time since diagnosis, time between the end of therapy and blood sampling, cranial irradiation, relapse risk at diagnosis, years of DFCI protocol, corticoid dosage, and doxorubicin dosage. These factors were used to predict the extent of immunoage gain, which served as the dependent variable in our analysis for cALL survivors. Subsequently, we conducted an analysis that involved the simultaneous inclusion of all these independent covariates. We then selected the relevant variables to form the final model. Tests of statistical significance were two-tailed, and *p* values of <0.05 were considered significant unless otherwise specified. Statistical analyses were performed using SPSS version 26 (IBM Corp., Armonk, NY, USA).

## Results

### Characteristics of the participants

This study comprised 248 survivors of cALL and 281 healthy controls (Table 1). The female:male ratio was 1:1 for cALL survivors and 1:1.15 for healthy controls. There was no statistical difference between the age of male (22.5 ± 6.2 years) and female (22.0 ± 6.5 years) cALL survivors. The standardized follow-up medical evaluation of cALL survivors occurred 3.3–26.1 years posttreatments. The prevalence of metabolic syndrome among the cALL survivors was 9.0% (n = 22). Reflecting overall health, the performance on the 6MWT was below the predicted value in 37.9% of survivors. A proportion of 10.3% of cALL survivors had hypothyroidism.

**Table 1:** Sociodemographic and clinical characteristics of participants.

	PETALE (cALL survivors) *n* = 248	CHUM (control 1): age 19–40 years old, *n* = 47	CARTaGENE (control 2): age 40–69 years old, *n* = 234
Sex (all participants)			
Males (n, %)	124; 50.0	14, 29.8	136, 56.9
Females (n, %)	124; 50.0	33, 70.2	98, 41.0
Age at diagnosis, mean in years (s.d.)	6.5 ( ± 4.5)	NA	NA
Age at biomarker sampling, means in years (s.d.)	22.3 ( ± 6.3)	29.1 ( ± 5.3)	54.5 ( ± 5.3)
Post-treatment period, mean in years (s.d.)	13.6 ( ± 5.3)	NA	NA
Irradiation exposure			
Yes (n, %)	149, 60.1	NA	NA
No (n, %)	99, 39.9		
Relapse risk group at diagnosis			
SR (n, %)	111, 44.8		
HR,cardioprotector (+) (n, %)	69, 27.8	NA	NA
HR,cardioprotector (−) (n, %)	60, 24.2		
Metabolic syndrome			
All (n, %)	22, 9.1	NA	
Males (n, %)	12, 10.0		
Females (n, %)	10, 8.2		
Abnormal TSH (Hypothyroidism)			
All (n, %)	25, 10.3	NA	NA
Males (n, %)	10, 8.3		
Females (n, %)	15, 12.3		
Abnormal 6MWT			
All (n, %)	92, 37.9	NA	NA
Males (n, %)	45, 37.2		
Females (n, %)	47, 38.5		
Abnormal CPET			
All (n, %)	175, 82.2	NA	NA
Males (n, %)	97, 45.5		
Females (n, %)	78, 36.6		

*HR* high relapse risk at diagnosis, *NA* not applicable because control group participants are not assessed for the parameter or not treated with this treatment modality, *SR* standard relapse risk at diagnosis, *6MWT* 6-minute walk test, *CPET* Cardiopulmonary Exercise T.

### Premature thymic aging in cALL survivors

**Fig. 1:**
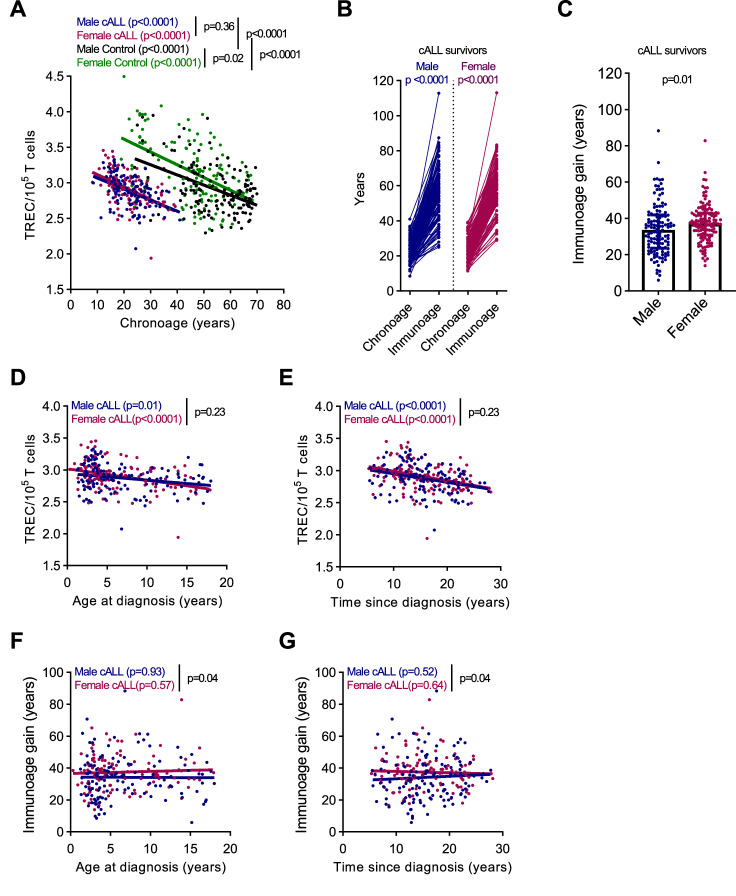
Accentuated thymic aging in cALL survivors is modulated by sex. **A** Correlation between chronological age and TREC levels. *p* values compare regression lines (slope and intercept). Pearson correlations: cALL males (*n* = 124, r = −0.356, R^2^ = 0.127; *p* < 0.0001) ; cALL females (*n* = 124; r = −0.501; R^2^ = 0.251; *p* < 0.0001); control males (*n* = 150; r = −0.466; R^2^ = 0.217; *p* < 0.0001); control females (*n* = 131, r = −0.504, R^2^ = 0.254; *p* < 0.0001). **B** Each line represents the chronoage and immunoage (in years) of one survivor; *n* = 124 males and 124 females. *p* values calculated using Wilcoxon test. **C** Comparison of immunoage gain in male and female cALL survivors. *p* values calculated using Mann–Whitney test; *n* = 124 females; *n* = 124 males. Correlation between TREC (**D**) or immnuoage gain (**F**) and age at diagnosis. Correlation between TREC (**E**) or immnuoage gain (**G**) and time elapse since diagnosis.

To determine the normal rate of T-cell compartment immunoaging we measured blood TREC levels normalized to the T-cell count in the Quebec reference population and observed a highly significant correlation with chronological age for both men and women (Pearson correlation: males, r = −0.466; females, r = −0.504; *p* < 0.0001) (Fig. 1A, controls). As reported in other populations, TREC levels were higher in healthy females than in healthy males, indicating the necessity to adjust for sex (Fig. 1A) [6]. To assess the sensitivity and reliability of TREC as an age-associated immunosenescence biomarker, intraindividual variation in TREC levels were compared at two paired time points in the control group: initial time (T0 = participant age X years old) and at a 6-year follow-up (T6 = participant age (X + 6) years old (average of 6.2 years ±0.5)). TREC levels at time T0 and T6 followed the expected age-associated decreasing trends (Supplementary Fig. 1A). Importantly, a paired intraindividual analysis between times T0 and T6 significantly tracked immune aging (Supplementary Fig. 1B), validating the sensitivity and reliability of a PCR TREC measurement to assess a 6-year increment in thymic aging. TREC measurements from the same blood sample were also highly reproducible (Supplementary Fig. 1H–I), suggesting that the interindividual variability observed in TREC levels reflect real biological changes.

We then evaluated immunosenescence using TREC levels, which were lower in cALL survivors than in sex-matched controls (*p* < 0.0001) (Fig. 1A). Unlike the controls, no sex differences in TREC levels were observed in cALL survivors (Fig. 1A, cALL). We ruled out that the lower levels of TREC in cALL patients resulted from peripheral dilution, because the total lymphocyte counts were comparable and in the normal range for cALL survivors and controls (Supplementary Fig. 1C) [36]. Overall, TREC levels were lower in cALL patients than in the normally aging cohort, while the rate of TREC loss per year was not significantly different between the cohorts (TREC decreased by 3.23% and 4.05% for male and female controls, respectively and 2.64% and 3.71% for male and female cALL survivors, respectively).

We extrapolated the immunoage of cALL survivors to portray the clinical size effect. In brief, we determined the immunoage for each cALL survivor by calculating the slope generated from plotting TREC levels against the chronological age (chronoage) of control participants (from the pooled CHUM and CARTaGENE cohorts). The results indicated a major shift in immunoage compared with chronoage for male and female cALL survivors (Fig. 1B). We defined years of immunoage gain as the difference between immunoage and chronoage for each individual; higher immunoage gain was observed for female (mean ± sd 37.4 ± 10.7) cALL survivors than for their male counterparts (mean ± sd 34.2 ± 14.0) (Fig. 1C).

Given their connection to age, TREC levels were also associated with age at diagnosis (Fig. 1D) and time elapsed since diagnosis (Fig. 1E) in male and female cALL survivors. However, there was no statistically significant association between these parameters and the extent in immunoage gain (Fig. 1F, G). Together, with the similar rate of TREC loss per year observed between the control and PETALE cohorts, these findings suggest that all cALL survivors first display a rapid initial acceleration in immunoage early during or after treatment followed by gradual thymic functional senescence, at a rate similar to that observed during normal aging resulting in overall continued accentuated immunoage. Decreased TREC levels were not linked to ALL subtype, because thymic aging and immunoage gain were similar for survivors affected by B-lymphocyte ALL (pre-B ALL) or T-lymphocyte (pre-T ALL) and immunoage gain did not change over time after diagnosis (Supplementary Fig. 1D–G).

Given a systematic immunoage gain in the thymic compartment of cALL survivors, we investigated whether regulatory cytokines related to T cells development and/or aging were abnormally altered in cALL survivors blood. We quantitatively measured circulating levels of each factors using quantitative multiplex ELISA and evaluated the relationship between IL-4, IL-6, IL-7, IL-12p70, IL-15, bFGF, GM-CSF, INFy, age, thymic output (TREC) and immunoage gain. Overall, no circulating factors were universally correlated both in males and females, but increased IL-6, IL-7, and IL-15, which are known to stimulate T cell proliferation or function, were associated with immunoage gain in the larger cohort (Table 2 and Supplementary table 1).

**Table 2:** Correlations and odds ratios of clinical characteristics, treatment modalities and T-cells related factors for chronoage, TREC and immunoage gain.

Clinical characteristics and treatment modalities	All survivors			Males			Females		
	Chronoage	TREC	Immunoage gain	Chronoage	TREC	Immunoage gain	Chronoage	TREC	Immunoage gain
Metabolic syndrome (positive, *n* = 10 males & 12 females) OR (95% CI)	1.107 (1.035–1.184) ***p*** = **0.003**	0.032 (0.004–0.235) ***p*** < **0.001**	1.038 (1.005–1.071) ***p*** = **0.02**	1.075 (0.978–1.182) *p* = 0.14	0.102 (0.007–1.428) *p* = 0.09	1.025 (0.986–1.066) *p* = 0.21	1.140 (1.034–1.256) ***p*** = **0.008**	0.oo7 (0.000–0.216) ***p*** = **0.005**	1.063 (1.006–1.123) ***p*** = **0.03**
Hypothyroidism (positive, *n* = 10 males & 15 females) OR (95% CI)	0.778 (0.692–0.874) ***p*** < **0.001**	16.549 (2.128–128.71) ***p*** = **0.007**	0.990 (0.957–1.024) *p* = 0.56	0.801 (0.681–0.942) ***p*** = **0.007**	82.747 (2.237–3060.95) ***p*** = **0.017**	0.960 (0.908–1.014) *p* = 0.15	0.758 (0.640–0.898) *p* = **0.001**	5.986 (0.467–76.78) *p* = 0.17	1.012 (0.964–1.063) *p* = 0.63
Abnormal 6MWT (positive, *n* = 45 males & 47 females) OR (95% CI)	1.044 (1.002–1.089) ***p*** = **0.04**	0.543 (0.170–1.736) *p* = 0.30	1.002 (0.981–1.022) *p* = 0.88	1.071 (1.006–1.139) ***p*** = **0.03**	0.614 (0.116–3.248) *p* = 0.57	0.996 (0.970–1.022) *p* = 0.75	1.023 (0.966–1.083) *p* = 0.44	0.469 (0.091–2.414) *p* = 0.37	1.01 (0.977–1.045) *p* = 0.55
Global deconditioning, (positive, *n* = 97 males & 78 females) OR (95% CI)	0.985 (0.932–1.041) *p* = 0.59	0.266 (0.050–1.415) *p* = 0.12	1.018 (0.989–1.048) *p* = 0.23	0.938 (0.950–1.035) *p* = 0.20	1.402 (0.092–21.47) *p* = 0.81	1.006 (0.963–1.052) *p* = 0.78	1.003 (0.936–1.073) *p* = 0.94	0.115 (0.012–1.088) *p* = 0.06	1.055 (1.004–1.108) ***p*** = **0.03**
Dose of doxorubicin, *n* = 242 (121 males & 121 females)	r = 0.335 ***p*** < **0.001**	r = −0.154 ***p*** = **0.02**	r = 0.012 *p* = 0.86	r = 0.458 ***p*** < **0.001**	r = −0.200 ***p*** = **0.03**	r = 0.026 *p* = 0.78	r = 0.222 *p* = **0.01**	r = −0.110 *p* = 0.23	r = −0.001 *p* = 0.99
Cranial irradiation dose (Gy), *n* = 246 (123 males & 123 females)	r = 0.418 ***p*** < **0.001**	r = −0.169 ***p*** = **0.008**	r = −0.046 *p* = 0.47	r = 0.497 ***p*** < **0.001**	r = −0.174 ***p*** = **0.054**	r = −0.027 *p* = 0.77	r = 0.341 *p* < **0.001**	r = −0.147 *p* = 0.10	r = −0.034 *p* = 0.71
Total corticosteroids, *n* = 242 (120 males & 122 females)	r = 0.358 ***p*** < **0.001**	r = −0.165 ***p*** = **0.01**	r = −0.007 *p* = 0.92	r = 0.394 ***p*** < **0.001**	r = −0.118 *p* = 0.20	r = −0.037 *p* = 0.69	r = 0.264 ***p*** = **0.003**	r = −0.188 ***p*** = **0.04**	r = 0.064 *p* = 0.49
Risk group (high-risk=positive, *n* = 66 males & 67 females), OR (95% CI)	1.121 (1.07–1.174) ***p*** < **0.001**	0.341 (0.107–1.082) *p* = 0.07	0.995 (0.976–1.015) *p* = 0.65	1.174 (1.091–1.265) ***p*** < **0.001**	0.284 (0.054–1.489) *p* = 0.14	0.995 (0.970–1.021) *p* = 0.72	1.081 (1.019–1.148) ***p*** = **0.01**	0.400 (0.079–2.022) *p* = 0.27	0.995 (0.963–1.029) *p* = 0.77
Cranial Irradiation (yes=positive, *n* = 82 males & 67 females), OR (95% CI)	1.155 (1.097–1.216) ***p*** < **0.001**	0.263 (0.080–0.869) ***p*** = **0.03**	0.992 (0.972–1.012) *p* = 0.43	1.243 (1.135–1.362) ***p*** < **0.001**	0.195 (0.033–1.165) *p* = 0.07	0.996 (0.970–1.023) *p* = 0.79	1.10 (1.034–1.171) ***p*** = **0.003**	0.383 (0.076–1.941) *p* = 0.25	0.991 (0.959–1.025) *p* = 0.60
IL6, *n* = 246	r = 0.103, *p* = 0.11	**r** = −**0.191,** ***p*** = **0.003**	**r** = **0.170,** ***p*** = **0.008**	r = −0.017, *p* = 0.85	r = −0.060, *p* = 0.51	r = 0.096, *p* = 0.29	**r** = **0.227,** *p* = **0.01**	**r** = −**0.327,** *p* < **0.001**	**r** = **0.247,** *p* = **0.006**
IL7, *n* = 246	r = −0.050, p = 0.43	r = −0.116, *p* = 0.07	**r** = **0.141,** ***p*** = **0.027**	r = −0.115, *p* = 0.21	r = 0.048, *p* = 0.60	r = −0.002, *p* = 0.98	r = 0.006, *p* = 0.95	**r** = −**0.298,** ***p*** < **0.001**	**r** = **0.345,** ***p*** < **0.001**
IL15, *n* = 246	r = 0.085, *p* = 0.17	r = −0.099, *p* = 0.12	r = 0.074, *p* = 0.25	r = 0.167, *p* = 0.065	r = −0.081, *p* = 0.37	r = 0.016, *p* = 0.86	r = −0.007, *p* = 0.94	r = −0.157, *p* = 0.083	**r** = **0.188,** ***p*** = **0.037**
GM-CSF, *n* = 246	**r** = −**0.128,** ***p*** = **0.045**	r = −0.103, *p* = 0.11	**r** = **0.178,** ***p*** = **0.005**	r = −0.100, *p* = 0.27	**r** = −**0.195,** *p* = **0.03**	**r** = **0.237,** ***p*** = **0.008**	r = −0.154, *p* = 0.09	r = −0.011, *p* = 0.90	r = 0.109, *p* = 0.23
IL-4, *n* = 246	r = 0.120, *p* = 0.06	r = −0.047, *p* = 0.46	r = −0.006, *p* = 0.93	r = 0.03, *p* = 0.74	r = 0.068, *p* = 0.46	r = −0.046, *p* = 0.62	**r** = **0.213,** ***p*** = **0.02**	r = −0.154, *p* = 0.09	r = 0.054, *p* = 0.55
bFGF, *n* = 246	r = −0.050, *p* = 0.44	r = 0.006, *p* = 0.93	r = 0.002, *p* = 0.98	**r** = −**0.192,** ***p*** = **0.03**	r = 0.049, *p* = 0.59	r = −0.023, *p* = 0.80	r = 0.083, *p* = 0.36	r = −0.040, *p* = 0.66	r = 0.013, *p* = 0.89
INFy, *n* = 246	r = −0.108, *p* = 0.09	r = 0.029, *p* = 0.65	r = 0.038, *p* = 0.56	r = −0.054, *p* = 0.56	r = −0.002, *p* = 0.98	r = 0.013, *p* = 0.89	r = −0.128, *p* = 0.16	r = 0.041, *p* = 0.65	r = 0.050, *p* = 0.58
IL12p70, *n* = 246	r = −0.069, *p* = 0.28	r = 0.058, *p* = 0.36	r = −0.009, *p* = 0.89	r = −0.177, *p* = 0.051	r = 0.052, *p* = 0.57	r = 0.051, *p* = 0.58	r = 0.082, *p* = 0.37	r = 0.043, *p* = 0.64	r = −0.119, *p* = 0.19

*Chronoage* chronological age, *TREC* T-cell receptor excision circle, *IL* interleukin.

### cALL thymic functional senescence acceleration is in part related to DNA damage

**Fig. 2:**
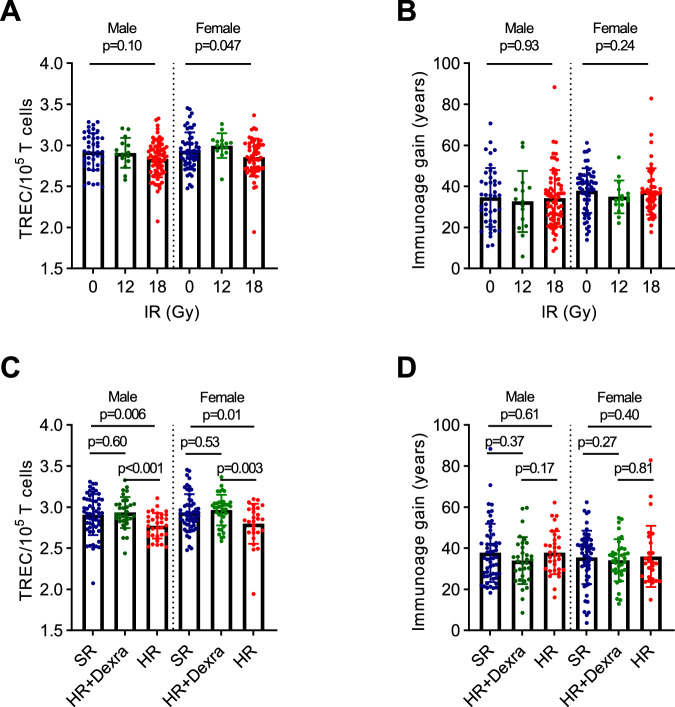
Thymic functional senescence accentuation is mostly treatment-decision independent. **A** Additional stratified analysis of sex and escalating total doses of cranial irradiation received associated with TREC levels. **B** Additional stratified analysis of sex and escalating total doses of cranial irradiation received did not associate with immunoage gain measured by extrapolating years of accentuated immunological aging. **C** Comparison of TREC levels by sex and by risk group. **D** Comparison of immunoage gain between risk groups by sex. **A**, **B**, n males (0 Gy = 42, 12 Gy = 15, 18 Gy = 67), n females (0 Gy = 57, 12 Gy = 15, 18 Gy = 52); (**C**, **D**), male (SR = 55, HR = 33, HR + dexra = 32), female (SR = 56, HR = 27, HR + dexra = 37). **B**, **D** Adjusted for age. No adjustment for other treatments is condered. SR standard risk, HR high risk, dexra: dexrazoxane. **A**–**D** *p* value by Mann–Whitney test.

DNA damaging agents such as radiation and doxorubicin cause cell senescence and thymic functional senescence [19, 37, 38]. However, the mecanism or clinical correlations between thymic aging in cancer survivors and their modulating factors have yet to be demonstrated. To verify whether accentuated thymic aging is linked to different DNA damages types in the context of cALL treatment, we tested its association with therapeutic modalities exposure. TREC levels decreased with high dose of cranial irradiation (Fig. 2A; Supplementary Fig. 2A), with corticosteroids in females and with high doses of doxorubicin in males (Table 2). Although females were more affected by corticosteroids or doxorubicin than males, none of the main treatment variations (i.e., cranial irradiation, corticosteroids, and doxorubicin) significantly influenced the magnitude of immunoage gain in cALL survivors (Fig. 2B; Table 2). Among patients in the high-risk of relapse at diagnosis category exposed to doxorubicin, those who received the cardioprotectant dexrazoxane had lower rates of thymic aging, because males and females exposed to higher doses of doxorubicin without cardioprotection showed an increase in thymic aging (i.e., decrease in TREC levels, Fig. 2C; Supplementary Fig. 2B). Despite these associations with TREC levels, immunoage gain was similar in all groups (Fig. 2D). This similarity could be due to sex-stratified treatments and analyses; for example, high doses of cranial irradiation in females (Fig. 2A) were associated with enhanced thymic aging as represented by TREC levels. Overall, through a multiple linear regression model, we found that only sex was a significant factor associated with immunoage gain in cALL survivors (Supplementary Table 2).

### Early-onset chronic diseases are associated with accentuated thymic functional senescence

**Fig. 3:**
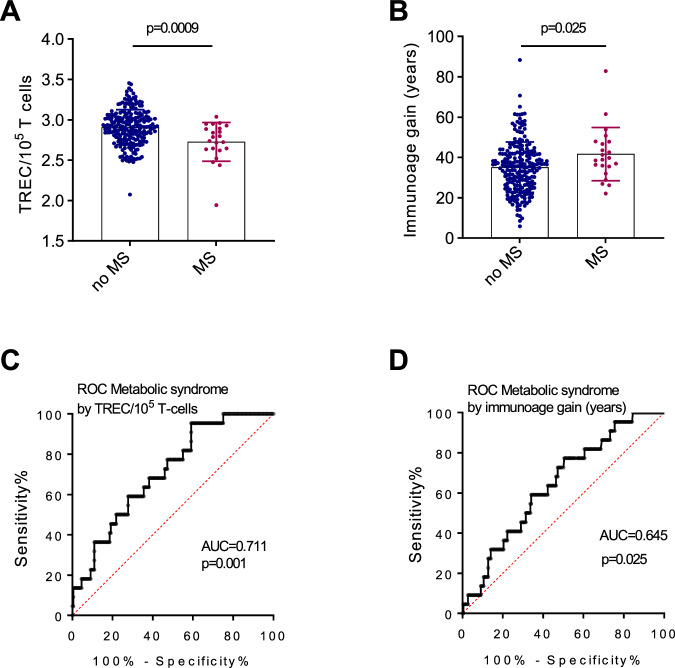
Accentuated thymic functional senescence is associated with metabolic syndrome. Association between thymic aging marker as measured by TREC levels (**A**) or years of immunoage gain (**B**) and metabolic syndrome status. **C** ROC curve of thymic aging marker as measured by TREC levels (**C**) and immunoage gain (**D**) as a metabolic syndrome incidence measure in cALL survivors (*n* = 22 MS; *n* = 220 no-MS). MS metabolic syndrome. The *p* value was calculated using the Mann–Whitney test.

To further define whether alterations in TREC loss or accentuated thymic aging are associated with enhanced susceptibility to early-onset chronic diseases in cALL survivors, we examined the association between chronological age, TREC levels, or immunoage gain, and clinical phenotypes. TREC levels were significantly associated with metabolic syndrome and hypothyroidism (Table 2). Notably, chronological age was also significantly associated with these clinical parameters because treatment selection for patients is partly age-dependent (Supplementary Fig. 2C, D). This finding was confirmed by the observation that immunoage gain, which is corrected by patient age, was seldom associated with the tested biochemical parameters (Table 2 and Supplementary Table 3). The single exception we observed was the association between accentuated immunological aging in all cALL survivors and metabolic syndrome. This association was notably stronger in female cALL survivors than in their male counterparts (Table 2). As we expected, the association was significant for TREC levels (Fig. 3A, Supplementary Fig. 3A) and for extrapolated immunoage gain in years (Fig. 3B, Supplementary Fig. 3B), while age at diagnosis did not alter the findings for metabolic syndrome (Supplementary Fig. 3B). We used ROC curves to test the diagnostic performance of low levels of TREC and high levels of immunoage gain to evaluate the presence of metabolic syndrome in cALL survivors. We found that accentuated thymic aging associated with metabolic syndrome onset with an accuracy of 71.1% for TREC levels (Fig. 3C) and 64.5% accuracy for immunoage gain (Fig. 3D). Analyzing the individual criteria for metabolic syndrome, we observed trends for blood systolic and diastolic pressure whose increase was accompanied by immunoage acceleration (Supplementary Table 3). This is also the case, to some extent, for increased body mass index, a risk factor for metabolic syndrome, which is associated with a significant decrease in TREC for both sexes but more heavily influenced by the chronological age of survivors (Supplementary Table 3). Moreover, in cALL survivors, immunoage gain increased (p = 0.02) as the body-fat percentage increased (Supplementary Table 3). Consistent with a connection between the thymic compartment and inflammation or T cell biology, increased IL-6 levels were also associated to metabolic syndrome (Table 3).

**Table 3:** Multiple linear regression for the association of T cells related cytokines and factors with metabolic syndrome as dependant variable.

Dependant variable	Metabolic syndrome			
covariates	Unadjusted, B (95% CI); *p*	Adjusted for age and sex	All covariates, B (95% CI); p	Selected covariates, B (95% CI); *p*
Chronoage	**0.009 (0.003 to 0.015),** *p* = **0.002**	**–**	**0.007 (0.001 to 0.013),** *p* = **0.02**	**0.008 (0.002 to 0.013),** *p* = **0.008**
Gender	−0.018 (−0.091 to 0.055), *p* = 0.63	–	−0.004 (−0.076 to 0.067), *p* = 0.91	−0.009 (−0.080 to 0.062), *p* = 0.81
IL6, *n* = 246	**0.143 (0.037 to 0.249),** *p* = **0.009**	**0.130 (0.025 to 0.236),** *p* = **0.016**	**0.160 (0.046 to 0.274),** *p* = **0.006**	**0.121 (0.014 to 0.228),** *p* = **0.027**
IL7, *n* = 246	−0.115 (−0.275 to 0.044), *p* = 0.16	−0.101 (−0.259 to 0.056), *p* = 0.21	−0.018 (−0.189 to 0.153), *p* = 0.84	–
IL15, *n* = 246	−0.225 (−0.648 to 0.198), *p* = 0.30	−0.269 (−0.688 to 0.150), *p* = 0.21	−0.168 (−0.632 to 0.296), *p* = 0.48	–
GM-CSF, *n* = 246	−0.011 (−0.097 to 0.076), *p* = 0.80	0.005 (−0.080 to 0.091), *p* = 0.90	0.001 (−0.087 to 0.088), *p* = 0.99	–
IL-4, *n* = 246	**0.152 (0.005 to 0.300),** *p* = **0.04**	0.135 (−0.011 to 0.281), *p* = 0.07	0.101 (−0.050 to 0.252), *p* = 0.19	0.114 (−0.034 to 0.262), *p* = 0.13
bFGF, *n* = 246	−0.033 (−0.145 to 0.079), *p* = 0.56	−0.026 (−0.136 to 0.084), *p* = 0.64	−0.045 (−0.156 to 0.066), *p* = 0.43	–
INFy, *n* = 246	−0.049 (−0.113 to 0.016), *p* = 0.14	−0.038 (−0.102 to 0.026), *p* = 0.24	−0.052 (−0.122 to 0.019), *p* = 0.15	–
Il-12p70, *n* = 246	−**0.152 (**−**0.303 to 0.00),** *p* = **0.050**	−0.134 (−0.285 to 0.016), *p* = 0.08	−0.140 (−0.292 to 0.013), *p* = 0.07	**−0.157 (−0.306 to −0.008),** *p* = **0.038**

Hypothyroidisms was associated with an increasing trend in thymic aging, revealed by decreased TREC levels and increased immunoage gain (Supplementary Fig. 3C, D). Knowing that thymus size can be affected by sex steroids, we tested and observed that increased levels of dehydroepiandrosterone sulfate (DHEAS), a sex hormone involved in the production of testosterone and estrogen, was significantly associated with increased immunoage gain in females (Supplementary Table 3) [4, 39].

### Global physical condition and accentuated thymic aging are not associated

**Fig. 4:**
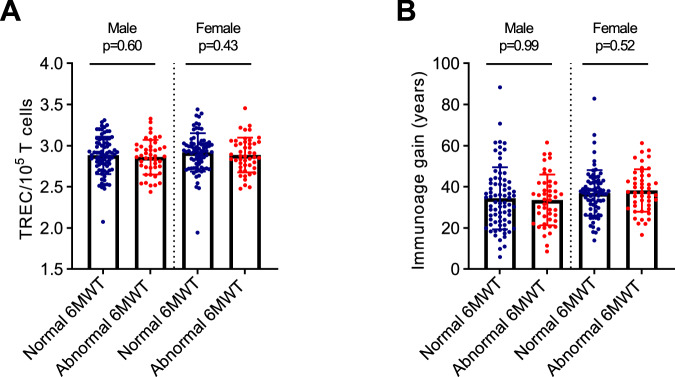
Accentuated thymic aging is not associated with the global physical condition. **A** Comparison by sex of TREC levels depending on whether cALL survivors have normal 6MWT, using Welch’s *t* test. **B** Comparison by sex of immunoage gain (years) depending on whether cALL survivors have normal 6MWT using Mann-Whitney test.

Frailty generally refers to unhealthy aging and can be defined using various pathophysiological parameters [16, 40, 41]. Several frailty indices include abnormally slow walking speed as part of their criteria [16, 40]. Thus, we used the 6MWT to assess aerobic capacity and endurance. Although associated with chronological age, we observed no association between the 6MWT results and immunoage gain or TREC levels in sex stratified analyses (Table 2, Fig. 4A, B). Perhaps indirectly related to both metabolic syndrome and global physical condition, female cALL survivors global deconditioning (exercise tolerance and sedentary) was also significantly associated to immunoage gain (Table 2).

## Discussion

In this study, we show for the first time that TREC levels are abnormally low for every participant of a cALL survivor cohort. We observed that the extent of this systematic premature thymic functional senescence averaged 37.4 years for females and 34.2 years for males. We also noted that the inflammation and T cell biology factors IL-7, IL-6, and GM-CSF, along with age, were associated with thymic compartment aging (Supplementary Table 1). Our results support an en bloc, systematic shift in immunological age for cALL survivors. We hypothesize that after being shocked by anticancer treatments, possibly thymic activity resumes its aging at an older state than expected for the patient’s age but without further accelerated degradation rate compared with what would normally be expected (graphical abstract). This is consistent with cytoablative agents damaging thymic epithelial cells and contributing to a prolonged impairment of thymopoiesis [21]. The extent of the accentuated thymic aging observed in cALL survivors using TREC measurement is in accordance with the 25–35 years of premature aging observed using the aging biomarker p16INK4a in peripheral blood T-lymphocytes from various childhood cancer survivors, but is higher than that evaluated using epigenetic age on blood DNA [27, 30]. Notably, TREC levels appear to follow a pattern similar to that proposed for telomere attrition, namely an initial rapid loss followed by further gradual attrition over time as in normal aging [28]. This phenomenon goes against the hypothesis that an inflection of epigenetic changes accumulation occurs at a stable rate from the time of treatment although both could occur following their independent clock [30].

It is well characterized that cALL survivors suffer from chronic inflammation and early-onset chronic diseases often associated with aging [12, 15]. Just as gradual changes in thymic output occur with age, deconditioning and compromised cardiopulmonary fitness are also considered manifestations of aging [42]. The independent aging rates of various tissues and organs have been extensively described in in vitro and animal studies [43–45]. Our observation that immunoage gain is associated with metabolic syndrome but not with global physical condition supports the hypothesis that accentuated aging in cancer survivor is perhaps segmental with specific aging biomarkers revealing specific morbidities. This highlight that no single aging biomarker or frailty score is likely to inform on the overall aging acceleration experienced by a cancer survivor. Accordingly, in the PETALE cohort, we find that chronological age and/or TREC levels are correlated with numerous physiopathological parameters including blood pressure, body mass index, blood creatinine, dehydroepiandrosterone (DHEAS), insulin-like growth factor 1, adiposity, and waist circumference. Upon correction for chronoage, which specifically isolate immunoage gain, a few factors remained significantly associated with TREC including metabolic syndrome, systolic and diastolic blood pressure and adiposity (Table 2 and Supplementary Table 3), suggesting these pathophysiological conditions could be particularly sensitive to thymic functional senescence regardless of chronological age.

We observed that females show greater accentuated aging than males, in agreement with a higher incidence of late adverse side effects in female cALL survivors than in their male couterparts [16, 27]. Thymic size is influenced by sex steroids and the hypothalamic-pituitary-adrenal axis [39, 46, 47]. Strikingly, an increase in female immunoage correlates with DHEAS levels, which can inhibit T-cell proliferation in the thymus before rearrangement leading to TREC excision [48]. The overall exact molecular and cellular mechanisms underlying accentuated aging among cALL survivors remain unclear, but our findings suggest thymic functional senescence as a component of it for the first time. Intertwined aging processes associated with stress adaptation, environmental exposures and life habits probably affect post-treatment aging via cell senescence, epigenetic modifications, chronic inflammation, macromolecular damages, disturbed metabolism, proteostasis, and stem-cell regeneration capacities [15, 49]. Deciphering the premature cALL aging mechanisms remains key to create clinical actionable targets. For example, targeted senescent cell elimination reduces the impact of chemotherapy-induced premature aging in animals and has been proposed as a promising strategy to attenuate age-associated organ dysfunctions [44, 50, 51].

Some of these late effects, including metabolic syndrome, obesity, and weight gain, are more frequent in cALL survivors who received therapy involving cranial irradiation and corticosteroids [12, 52]. Additionally, cranial irradiation can lead to thyroid dysfunction and is associated with increased risk of hypothyroidism [12, 53]. Younger age at diagnosis and the time elapsed since diagnosis are also typically associated with higher risks of late adverse effects [54]. However, we did not observe any association between age at diagnosis and decrease in thymic output. Nonetheless, even though naïve T cells do not accurately reflect the levels of RTE due to peripheral proliferation compensating for it, other authors have demonstrated that after chemotherapy, younger individuals experience more robust recovery within the first four years [19]. While previous research has described the impact of treatments such as doxorubicin and irradiation on thymic function, TREC measurements in this context have not been conducted [21, 22, 38, 55, 56]. In accordance with the general idea, both TREC and chronological age in PETALE are strongly correlated with similar treatment-modality parameters (Table 2). Interestingly, treatment decisions in cALL are highly dependent on patient age, and following chronoage correction, we did not observe a direct association of immunoage gain with treatments. In addition to the strong link between patient age and treatment selections, this lack of association may be attributed to the time gap between cALL therapy and the time of sampling (mean±sd, 13.6 ± 5.3 years), or conversely because the survivors are not yet old enough to develop further age-associated pathologies. We also cannot rule out the potential influence of a combination of factors that may require the analysis of a larger patient cohort, for example treatment modalities (doxorubicin, radiation, etc.), ALL disease and relapse risk groups.

Specifics cytokines play a role in T cells development, for example intrathymic or systemic production of GM-CSF, IL-6 and IL-7 regulate thymopoietin and thymus involution [57]. This is consitent with the association we report between immunoage gain (premature aging) and increase in these cytokines (Table 2), and with prior reports of a negative correlation betwen IL-6 and TREC levels [57]. Despite the correlations between TREC and cytokines, it remains challenging to determine the cause-and-effect relationship. These factors could be responsible for the reduction in RTE production (as revealed by TRECs), just as the decrease in thymopoiesis could also be the cause of the increased systemic cytokine secretion by senescent cells [1, 2]. Factors like GM-CSF, IL-1, IL-6, TNF-α, and IFN can also drive the differentiation of hematopoietic stem and progenitor cells towards myeloid cell creating an imbalance in lymphopoiesis [58]. Despite the potential stimulatory function of IL-12 on thymocyte proliferation through increased IL-7 and IL-2 signaling as well as its enhancement of hematopoietic reconstitution after transplantation, we did not observe any connections [3]. Interestingyl IL-7 correlated inversely with total lymphocytes count (r = −0.198, *p* = 0.002), consistent with a prior study in individuals experiencing lymphopenia [21].

Among the plasma factors associated with T-cell development, we observed that the inflammatory factors IL-6 and IL-12p70, along with age, are linked to metabolic syndrome. The associations between TREC, immunoage, IL-6, and metabolic syndrome suggest a potential state of greater thymic functional senescence for some survivors, at least in a segmental manner for the thymic compartment. This provides a potential target for mitigating the late effects of cALL survivors by targeting IL-6 or its mechanisms of induction.

Since thymus atrophy measured using TREC levels is an independent predictor of all-cause mortality in healthy elderly humans, the implications of our results for cALL survivors remain concerning [59, 60]. Because only recently a high percentage of patients with cALL survived into adulthood, the lifelong implications of this accentuated thymic aging remain unclear. In our cALL survivors cohort, accentuated thymic aging was associated with early-onset metabolic syndrome in young population but not with other physical conditions (for now). Indeed, because members of our cohort were relatively young at study time (22.3 years old +/−6.3), we are expecting more associations between immunoage gain and early-onset diseases as the participant’s age and the incidence of chronic diseases increases.

Additional studies in older survivors cohorts, including longitudinal studies, are needed to determine how TREC measurement could perhaps integrate a follow-up screening biomarker panel to identify cALL survivors at high-risk of developing chronic diseases such as metabolic syndrome. Major advantages of TREC measurement over previous premature aging biomarkers in cancer survivors are its fastness, reproducibility (Supplementary Fig. 1H, I), non-invasiveness, cost effectiveness, while requiring a minimal amount of blood DNA without particular preservation techniques (<100 μl whole blood fresh or frozen) [4]. In short TREC measurement could facilitate the quantitative molecular assessment of premature immunological aging in comparison to classical aging biomarkers, such as T-cell p16INK4a RNA expression, telomere length or methylation clock, which are measures sensitive to blood component degradation or require specialized sample preparation, preservation and analysis that are complicated to implement in a routine clinical setting [4, 26, 28, 30].

Despite the systematic and very significant changes in TREC levels observed in cALL survivors, several limitations and constraints should be considered in interpreting TREC as a pathology prediction biomarker. These limitations include small sample size, missing data, no temporality difference between the thymic aging biomarkers and outcomes and cross-sectional monocentric design. Additionally, we were unable to recruit age-matched participants under 19 years of age to match the 8–18 year-old survivors and unlike for TREC we have not obtained control samples for circulating factors measurement during normal aging. Furthermore, since our findings are specific to the cALL survivors, they cannot be extrapolated to adult-onset cancer survivors or non-hematopoietic cancers survivors.

In summary, we found that TREC measurement in cALL survivors is a biomarker of premature thymic aging, which is, correlated to early-onset metabolic syndrome and some inflammatory biomarkers. Pending validation in other large cohorts, we suggest that TREC levels could function as a biomarker that could be used to complement or integrate into cALL survivors’ frailty assessments. TREC levels offer the benefits of simplicity, cost-effectiveness, an accessible laboratory test and rapid analysis. A longitudinal study that measures concomitantly this marker with well-known one (frailty score, telomere length or epigenetic clocks, senescence markers) and monitors the occurrence of health decline and diseases would be beneficial in establishing its added value in the follow-up of this population. Finally, animal models and exploration of other human cancer survivor cohorts could be beneficial in improving our comprehension of the biological mechanism that results in post-treatment changes in TREC levels.

### Supplementary information

SUPPLEMENTAL DATA

Supplementary table 3

## Data Availability

The data will be available upon request (contact rodierf@mac.com) and can be sent by email to the corresponding author.
