# Supplementary material for: Premature thymic functional senescence is a hallmark of childhood acute lymphoblastic leukemia survivorship
Source: Blood Cancer J. 2024 Jun 13;14(1):96. doi: 10.1038/s41408-024-01071-1 (PMC11176394; doi:10.1038/s41408-024-01071-1)
Supplement: Supplementary file 1 — SUPPLEMENTAL DATA [file 41408_2024_1071_MOESM1_ESM.pdf]

SUPPLEMENTAL DATA

Supplementary methods

TREC quantification

Genomic DNA extraction and copy-number plasmid controls

Genomic DNA was extracted from peripheral blood mononuclear cells (PBMC) or whole blood samples using the PureLink® Genomic DNA Minikit (Thermo Fisher, USA) according to the manufacturer's protocol. CD3g (Accession: NG_007566), VDJ (Accession: NG_001333). TREC (Accession: NC_000014) amplification target sequences were synthetized in two pMA-t plasmid vectors using GeneArt gene synthesis according to the manufacturer's protocol (ThermoFisher, USA). These plasmids were linearized using the XhoI restriction enzyme and used as copy number standards in PCR reactions. Vector sequences are available in Supplementary Table 4.

Preamplification and multiplex Real-time quantitative PCR (qPCR):

Copy number standards and DNA samples were preamplified in the same conditions in 10 µl reaction volume composed of 25 nM of each TREC preamplification primer, the mix and 0.2 to 0.5 µg of gDNA. The master mix included a PCR buffer (1x), dNTP (200 µM), $MgCl_2$ (1.5 mM) and Taq (10 U/mL) provided by Invitrogen® (USA). Amplification was done using a Biometra T3 Thermocycler (Göttingen, Germany) at 94°C for 3 min., 15 cycles (94°C for 3 min, 55°C for 30 sec and 72°C for 90 sec) and 72°C for 10 min. The preamplified DNA was diluted 1/6 in Dnase/Rnase free water before multiplex qPCR. The total reaction volume per well was 10 µl, including diluted preamplified DNA, PCR master mix, primers and probes for the three target sequences (Supplementary Table 5). The TaqMan™ Fast Advanced Master Mix was purchased from Thermo Fisher Scientific (Lithuania) and the primers and probes from Integrated DNA Technologies (USA). We perform triplex quantitative multicolor TaqMan™

PCR amplification of TREC, CD3 and unrearranged VD-J in a single tube, each time normalized using a dilution-to-single-copy reference plasmid containing all three amplicons. This was done in triplicate using the StepOne Plus^{TM} real time system (Applied Biosystem, California, USA) according to the following protocol: 50°C for 2 min, 95°C for 10 min, followed by 40 cycles of 95°C for 15 sec and 60°C for 1 min. Number of TREC copies were evaluated for each sample normalized to T cells number. Briefly, TREC value estimated in ratio of TREC by 100 000 T cells. T cells count were obtained by subtracting total blood cells that are not T-cells (unrearranged VD-J copy number divided by 2) from the total nucleated white blood cells (CD3 copy number divided by 2).

Supplementary Tables

Supplementary table 1 : Multiple linear regression for the association of plasma factors with T cell development or thymus function

Dependent variable / Covariates	Unadjusted, B (95% CI); p		All covariates, B (95% CI); p	
	**TREC(Log)**	**immunoage gain**	**TREC(Log)**	**immunoage gain**
**Chronoage, n=248**	**-0.017 (-0.021 to -0.13), p=1.2x10^{-15}**	0.033 (-0.216 to 0.281), p=0.80	**-0.017 (-0.021 to -0.013), p=2x10^{-15}**	0.051 (-0.200 to 0.302), p=0.69
**Gender, n=248**	0.033 (-0.023 to 0.089), p=0.25	**3.243 (0.126 to 6.360), p=0.04**	0.025 (-0.024 to 0.073), p=0.32	**3.214 (0.042 to 6.309), p=0.04**
**IL6, n=246**	**-0.140 (-0.221 to -0.060), p=0.0007**	**6.740(2.180 to 11.299), p=0.004**	**-0.117 (-0.194 to -0.040), p=0.003**	**6.703 (1.792 to 11.615), p=0.008**
**IL7, n=246**	-0.113 (-0.235 to 0.009), p=0.068	**7.699 (0.882 to 14.515), p=0.027**	**-0.130 (-0.245 to -0.014), p=0.03**	6.512 (-0.875 to 13.899), p=0.08
**IL15, n=246**	-0.286 (-0.609 to 0.037), p=0.08	14.787 (-3.346 to 32.921), p=0.11	-0.033 (-0.346 to 0.281), p=0.84	2.424 (-17.608 to 22.456), p=0.81
**GM-CSF, n=246**	-0.052 (-0.118 to 0.014), p=0.12	**5.386 (1.731 to 9.042), p=0.004**	**-0.068 (-0.128 to -0.009), p=0.02**	**4.824 (1.034 to 8.614), p=0.01**
**IL-4, n=246**	-0.040 (-0.153 to 0.074), p=0.49	-0.463 (-6.862 to 5.936), p=0.89	-0.023 (-0.079 to 0.125), p=0.66	-2.284 (-8.818 to 4.249), p=0.49
**bFGF, n=246**	-0.009 (-0.094 to 0.077), p=0.84	0.983 (-3.824 to 5.791), p=0.69	-0.001 (-0.076 to 0.074), p=0.97	0.041 (-4.747 to 4.828), p=0.99
**INFy, n=146**	0.003 (-0.046 to 0.053), p=0.89	1.212 (-1.575 to 3.999), p=0.39	-0.020 (-0.027 to 0.068), p=0.40	-1.185 (-4.224 to 1.854), p=0.44
**Il-12p70, n=246**	0.054 (-0.063 to 0.171), p=0.36	-0.470 (-7.029 to 6.089), p=0.89	0.050 (-0.053 to 0.153), p=0.34	-2.584 (-9.162 to 3.995), p=0.44

Supplementary table 2 : Linear regression model of sociodemographic and clinical factors association to immunoaging in cALL survivors

factors	Survivors, n	Unadjusted<br>B (95% CI); p	All covariates<br>B (95% CI); p	Selected covariates<br>B (95% CI); p
**Chronoage**	248	0.033 (-0.216 to 0.281); p=0.80	-	-
**Sex**	248	3.243 (0.126 to 6.360); p=0.04	3.263 (-0.024 to 6.55); p = 0.052	3.377 (0.219 to 6.535); **p = 0.04**
**Age at diagnosis**	248	0.026 (-0.322 to 0.373); p=0.88	0.029 (-0.383 to 0.441); p = 0.89	-
**Time since diagnosis**	248	0.031 (-0.266 to 0.328); p=0.84	-	-
**Time between end of therapy and blood sampling**	248	0.024 (-0.271 to 0.319); p=0.87	0.088 (-0.264 to 0.440); p = 0.62	-
**Cranial irradiation or not**	248	-1.273 (-4.478 to 1.932); p=0.44	-	-
**Dose of Cranial irradiation**	246	-0.069 (-0.255 to 0.118); p=0.47	-0.105 (-0.418 to 0.209); p = 0.51	-
**Relapse risk group at diagnosis**	247	-0.722 (-3.880 to 2.436); p=0.65	-8.083 (137.512 to -1.346); p = 0.09	-7.998 (-16.736 to 0.741); p = 0.07
**DFCI protocol**	245	-0.120 (-1.510 to 1.271); p=0.87	-	-
**Dose of corticoids (in prednisone-equivalents)**	242	$-1.04x10^{-5}$ (0.00 to 0.00); p=0.95	0.0001 (-0.0004 to 0.001); p = 0.62	-
**Dose of doxorubicin**	242	0.001 (-0.012 to 0.015); p=0.86	0.034 (-0.005 to 0.073); p = 0.08	0.033 (-0.004 to 0.069); p = 0.08

Y: immunoage gain.; X: independent variable ;

Linear regression model of sociodemographic and clinical factors association to immunoaging in cALL survivors is done using the latter as dependant variable.

Supplementary table 3 : Relationship between age, TREC and immunoage gain with clinical and biochemical parameters.

(Please see separately attached Excel file)

Supplementary table 4 : Vectors sequences:

**CD3/VDJ control vector sequence**	CTCGAGGAGTTTAAGAGGTTTAGCCACGCTACGGGGCTGCTGGGAGCCCGGCAGTCTGGCCACAGGAG<br>GTCGGTTTCACGGAAGGGCAGGATGTGGCGGCATCTCCTGAATTTAAGGAGTCTTGGGGGCGCGGTGCT<br>TCTCTGTCATTGGGCAACTCATTTTAGCCTCTTGGGGTTTCAGTTCCTCAACTGAGAACAAGGAATTTAG<br>GTTGAAATGAACATGCTGGAAAGCTTTCAAGAATTGCACACGTGAAATGCTCTTTGCGTGTCTCCCGGT<br>CTCCCACCCGCCCCCGACACAGAGGCGCAGGAGTAACCCTGCTCCCTTCCGCGTCCTCGCCCCACCACG<br>AGCTGCGCATTCTTCTCGCCCCCTCAAGTGGCCGAGCTCTCGAGTGGCTGGCTGGCTGCTAAGGGCTGC<br>TCCACGCTTTTGCCGGAGGACAGAGACTGACATGGAACAGGGGAAGGGCCTGGCTGTCCTCATCCTGGC<br>TATCATTCTTCTTCAAGGTAAGGGCCTACTAGGGGTCTGGAAGCCTGGGGAAGGGCTCAAGGGAAGAG<br>CCCATCACTAGTGAGACAGGAATATTGGTATCCCTAACCTTCAGCCTACCTCTGCTGTCACCTTAGAGTT<br>CAAAGAAGGGCAAAATGGAGGCTCTTAACTGTTCTCTGCTAGAGAGAAACAGTGTCCCATGGAGGAGA<br>AGGAATCCTTGTCTCTGAAAAATGCAAACAGAGTACTTAAATGGCTGAAGAGAGGACCCTGTTACCGCC<br>ATCTTAGATTTGAATGCAGCCCAAAAGGGCATAGGCCAAGAAACTAAAAGGAAAAAGTATATGTTCCC<br>TACTTCAGAGCTGGGGGCTAGCAGTCGACCTAGGAAATGTCCATTCACTCAGTTGGGCAGTTGGCTCGA<br>G
**TREC control vector sequence**	CTCGAGTGTCTTCATCCCTGAAATACACTCTGCTCTCTCCTATCTCTGCTCTGAAAGGCAGAAAGAGGGC<br>AGCCCTCTCCAAGGCAAAATGGGGCTCCTGTGGGGAACAGAGGGGTGCCTCTGTCAACAAAGGTGATG<br>CCACATCCCTTTCAATAGCACGTAGCCCAGAGGTGCGGGCCCCATCCTCTCGTGTGAGGAGCCCACGGT<br>GATGCATAGGCACCTGCACCCCGTGCCTAAACCCTGCAGCTGGCACGGGCCCTGTCTGCTCTTCATTCA<br>CCGTTCTCACGAGTTGCAATAAGTTCAGCCCTCCATGTCACACTGTGTTTTCCATCCTGGGGAGTGTTTC<br>ACAGCTATCCCAAGCCCCACGCTGACGAATCACGGCCGAAAACACACTCTGATGCCAGCACAGACCAC<br>GGAGCAAATGTCAGACAAGATCAGCCTCGGAAAAGTGAGTCCTCTCGAG

Supplementary table 5: Primers and probes

Reaction	Type	Sequence	Reference	Fluorophore
**Pre-amplification TREC**	TREC Fw	TCTCTCCTATCTCTCTGCTCTGCTG		
	TREC Rev	CTGACATTTGCTCCGTGGTC		
**qPCR Triplex (CD3, VDJ, TREC)**	TREC Fw	5’-CCTCTGTCAACAAAGGTGAT-3’	[1, 2]	
	TREC Rev	5’-GTGCTGGCATCAGAGTGTGT-3’	[1, 2]	
	TREC Probe	CACGGTGATGCATAGGCACCTGC	[3, 4]	JOE
	VD-J Fw	ACACGTGAAATGCTCTTTGCG	[5]	
	VD-J Rev	TTACTCCTGCGCCTCTGTGTC	[5, 6]	
	VD-J Probe	TCTCCCGGTCTCCCACCCGC	[5]	TAMRA
	CD3g Fw	GGCTATCATTCTTCTTCAAGGT	[7, 8]	
	CD3g Rev	CCTCTCTTCAGCCATTTAAGTA	[7, 8]	
	CD3g Probe	ATGGAGGCTCTTAACTGTTCTCTGCT	[8]	FAM

Supplementary figures

Supplementary figure 1

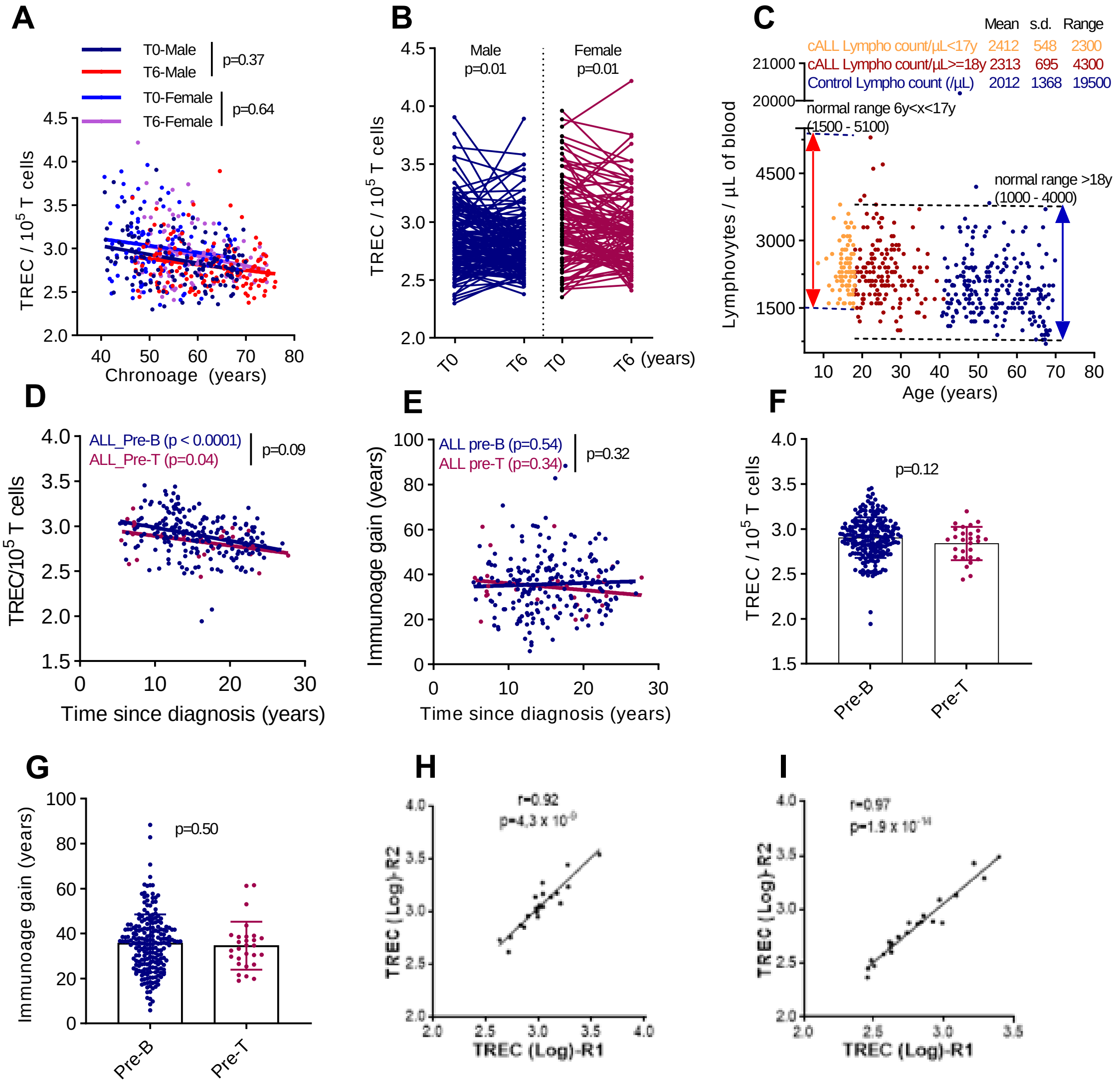

Supplementary figure 1: TREC levels decrease on a six years interval in the control cohort and are independent of leukemia subtype.

(A & B) comparison of immunoage (TREC) in control participant at two distinct time points on an average of six years interval. **(C)** Lymphocytes count in both population of cALL survivors and control by chronoage with normal range, (98.69 % of control and 97.98% of survivors are in normal range); $n_{control}$=229; n_{cALL}= 248. Immunoage in TREC (**D**) and immunoage gain in years (**E**) by sub-type of ALL according the time since diagnosis. (**F**) Comparison of immunoage in TREC by sub-type of ALL (unpaired t-test). (**G**) Comparison of immunoage gain (years) according to the sub-type of ALL (unpaired t-test). (**H**) Repeated measures in 2 different preamplifications followed by qPCR. (**I**) Repeated of two qPCR from the same preamplification. **(B)** t-test; $N_{control\text{-}male}$ (T0=T6=136); $n_{control\text{-}female}$ (T0=T6=98). **(A, D & E)** Statistic analysis is a Pearson correlation and comparison of slope and intercept was tested.

Supplementary figure 2

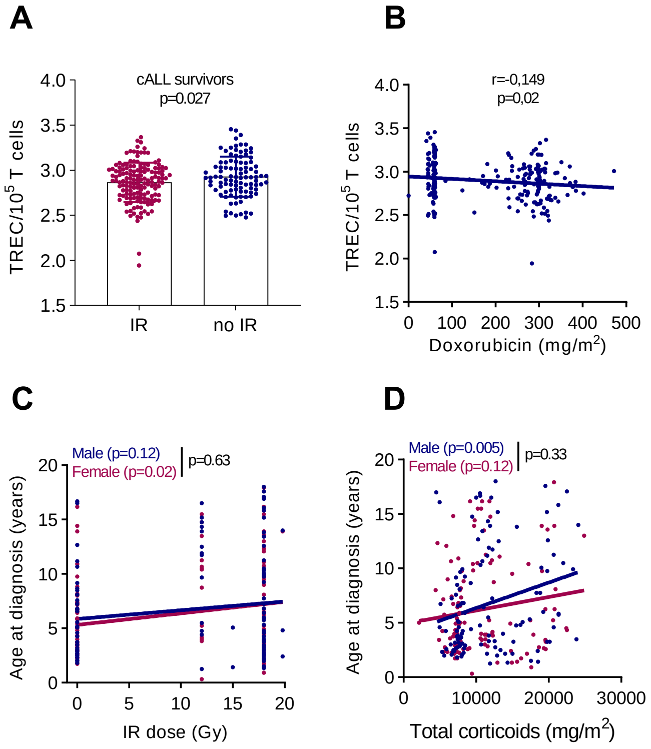

Supplementary figure 2: Immunosenescence marker (TREC) globally decrease with IR and doxorubicin among cALL.

(**A**) Comparison of immunoage (TREC) of cALL survivors between group having received IR or not (unpaired t-test). **B)** Correlation immunoage (TREC) and total dose of doxorubicin used in cALL therapy. Pearson correlation: n=246; r= -0.149, p=0.02. Pearson correlation: male (n= 121; r=0.253; p=0.005); female (n=122; r=0.143; p=0.12). Statistical analysis is a Pearson correlation and comparison of slope and intercept was tested. IR: cranial irradiation.

Supplementary figure 3

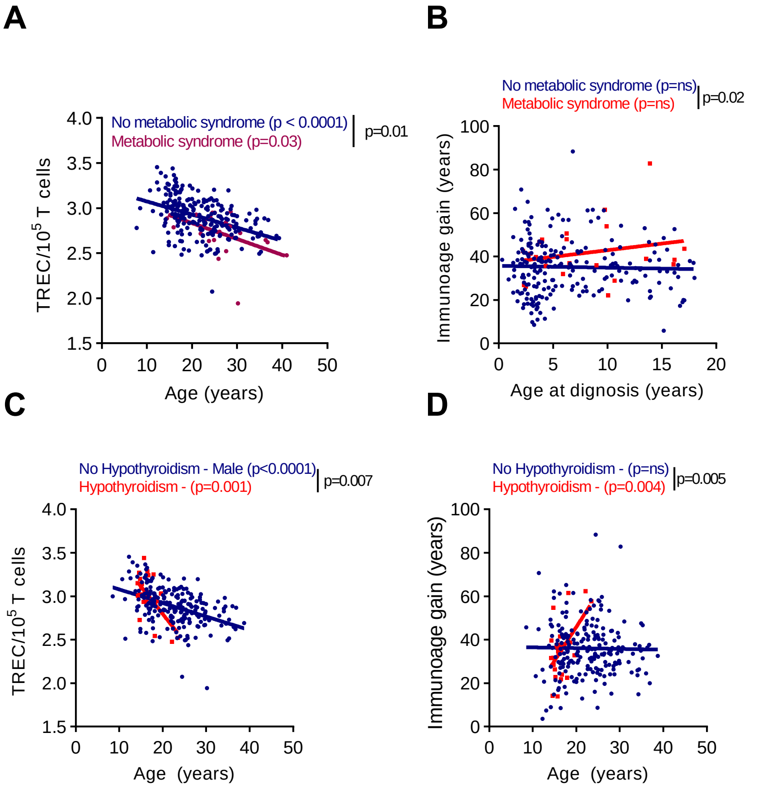

Supplementary figure 3: Increased immunosenescence is associated to an increase risk of inflammatory conditions.

(A) Comparison of immunoage (TREC) between survivors with or without metabolic syndrome according to their chronoage using linear regression. **(B)** Comparison of immunoage gain (years) by sex between survivors with or without metabolic syndrome according to their age at diagnosis using linear regression**.** **(C)** Comparison of immunoage (TREC) between survivors with or without hypothyroidism according their chronoage using linear regression. (**D**) Comparison of immunoage gain (years) between survivors with or without hypothyroidism according their chronoage using linear regression.

Statistical analysis is a Pearson correlation and comparison of slope and intercept was tested. **(A&B)** $n_{no\ metS}$= 221 & n_{metS}=22. **(C&D)** $n_{no\ hypothyroidism}$=215 & $n_{hypthyroidism}$=25).

Graphical abstract

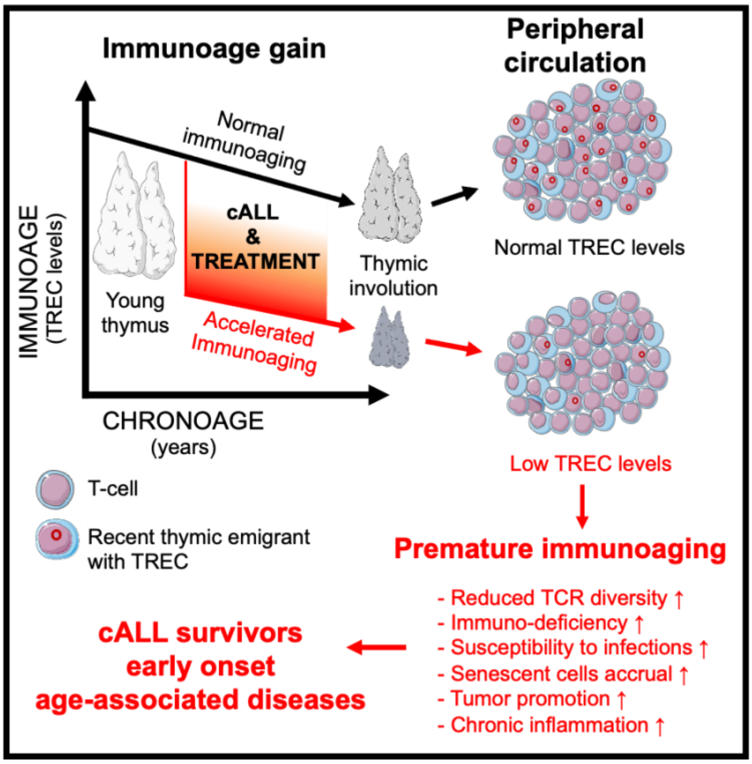

Graphical abstract: Accentuated immunoaging dynamics in cALL patients: cALL patients suffer an accentuated thymic atrophy around the time of cancer treatment leading to a rapid TREC loss followed by a gradual decline over time. TREC loss reflect reduced peripheral levels of recent thymic emigrants with TREC, which underlies multiple immunoaging phenotypes including low-level chronic inflammation, eventually leading to early-onset age-associated adverse health effects in cALL survivors. The images depicting T cells and the thymus in the graphical abstract have been adapted from Servier Medical Art (https://smart.servier.com), licensed under CC BY 4.0 (https://creativecommons.org/licenses/by/4.0/).

SUPPLEMENTARY REFERENCES

1. Dion, M.L., et al., *HIV infection rapidly induces and maintains a substantial suppression of thymocyte proliferation.* Immunity, 2004. **21**(6): p. 757-68.
2. Jacobson, J.M., et al., *Granulocyte-macrophage colony-stimulating factor induces modest increases in plasma human immunodeficiency virus (HIV) type 1 RNA levels and CD4+ lymphocyte counts in patients with uncontrolled HIV infection.* J Infect Dis, 2003. **188**(12): p. 1804-14.
3. Ou, X., et al., *Detection and quantification of the age-related sjTREC decline in human peripheral blood.* Int J Legal Med, 2011. **125**(4): p. 603-8.
4. Hazenberg, M.D., et al., *T cell receptor excision circles as markers for recent thymic emigrants: basic aspects, technical approach, and guidelines for interpretation.* Journal of molecular medicine, 2001. **79**(11): p. 631-640.
5. Chain, J.L., et al., *Real-time PCR method for the quantitative analysis of human T-cell receptor gamma and beta gene rearrangements.* J Immunol Methods, 2005. **300**(1-2): p. 12-23.
6. Lang, P.O., et al., *Real time-PCR assay estimating the naive T-cell pool in whole blood and dried blood spot samples: pilot study in young adults.* J Immunol Methods, 2011. **369**(1-2): p. 133-40.
7. Dion, M.L., R.P. Sekaly, and R. Cheynier, *Estimating thymic function through quantification of T-cell receptor excision circles.* Methods Mol Biol, 2007. **380**: p. 197-213.
8. Vandergeeten, C., et al., *Cross-clade ultrasensitive PCR-based assays to measure HIV persistence in large-cohort studies.* J Virol, 2014. **88**(21): p. 12385-96.
